# Conjugated Diphenylaniline-Azulene Co-Oligomers with Intense Absorption and Emission in the Visible Region

**DOI:** 10.3390/molecules29215041

**Published:** 2024-10-25

**Authors:** Nurlan Merkhatuly, Amantay Iskanderov, Saltanat Abeuova, Ablaykhan Iskanderov, Saltanat Zhokizhanova

**Affiliations:** 1 Laboratory of Organic Semiconductor Chemistry, Karaganda Buketov University, Karaganda 100028, Kazakhstan; 2 The Higher School of Natural Sciences, Astana International University, Astana 020000, Kazakhstan; abeuova.salta@gmail.com; 3 Department of Physics and Chemistry, Saken Seifullin Kazakh Agro Technical Research University, Astana 010011, Kazakhstan; saltanat_zh75@mail.ru

**Keywords:** azulene, diphenylaniline-azulene, conjugated azulene co-oligomers, cross-coupling, electron spectra, fluorescence

## Abstract

New conjugated 2,6-diphenylaniline-azulene co-oligomers of linear and branched structure were synthesized by the interaction of borylazulenes with diphenylaniline bromides under Suzuki–Miyaura cross-coupling conditions. The obtained diphenylaniline-azulene co-oligomers intensively absorb and emit visible light (410–700 nm region); in particular, they exhibit strong emissions in the green, as well as orange, range, with maxima of 510/590 nm. It is shown that such properties appear as a result of the positive resonance exposure to aniline fragments significantly rearranging the electronic structure of azulene, in particular, the levels and energy gaps of frontal HOMO–LUMO orbitals.

## 1. Introduction

New strategies for the development of functional materials are of the considerable interest in the dynamics of the development of electronic devices (organic light-emitting diodes, organic field-effect transistors, dye-sensitized organic solar cells, and others) [1,2].

Here, research focuses on the production and use of various molecules (building blocks) that are able to correctly debug the electronic structure of substances to improve and increase the performance of devices.

To date, the most effective molecules as building blocks are aromatic hydrocarbons, for example, azulenes.

Azulenes, as non-alternant aromatic compounds, have been the objects of a lot of scientific research over the past few decades [3,4,5,6,7,8,9,10,11].

Currently, azulene, due to its potential, is widely studied as a functional material for conducting oligomers and polymers, organic light-emitting diodes and other organic semiconductor devices [12,13,14,15,16,17,18,19,20,21,22,23].

Such interest in them was caused by the dipole structure (µ = 1.08 D) [24] and unusual optical properties, including S_2_→S_0_ anti-Kasha fluorescence [25,26,27,28,29].

**Figure 1:**
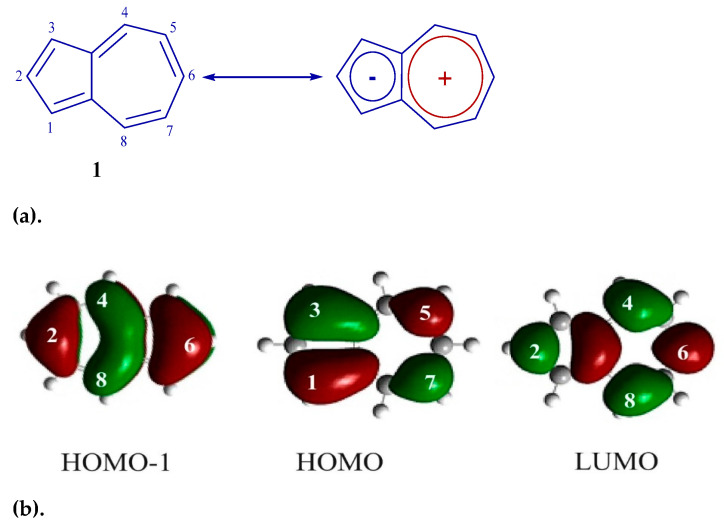
(**a**) Azulene **1** structure. (**b**) Frontal molecular orbitals **1**.

Azulene consists of negatively charged five-membered and positively charged seven-membered cycles (Figure 1a). This kind of structure gives high HOMO energy levels and low LUMO energies compared to conventional aromatic compounds [30,31,32,33,34]. In addition, the C-1 and C-3 carbon atoms have high HOMO ratios, and the C-2 and C-6 atoms have high HOMO-1 and LUMO ratios (Figure 1b) [30,31,32,33]. Azulene is also blue-colored compared to the colorless isomer naphthalene and shows absorption in the visible region of the spectrum at 580 nm (intensity is only 350 M^−1^ cm^−1^) caused by the forbidden transition S_0_→S_1_ [34].

Thus, it is expected that the introduction of donor diphenylaniline groups at the C-2 and C-6 positions of azulene can lead to a new advanced functional material.

In this work, we report the synthesis with high yields of new conjugated 2,6-diphenylaniline-azulene co-oligomers of linear and branched structures **6** and **8**, via the Suzuki−Miyaura reaction.

## 2. Results and Discussion

**Scheme 1:**
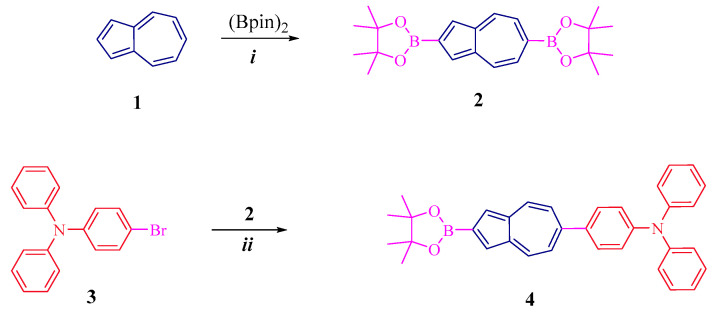
Synthesis **4**: (*i*) [IrCl(cod)]_2_, 2,2′-bipyridine, cyclohexane, reflux, yield 50%; (*ii*) Pd(PPh_3_)_2_CI_2_, THF:water (4:1), 75–80 °C, 70%.

**Scheme 2:**
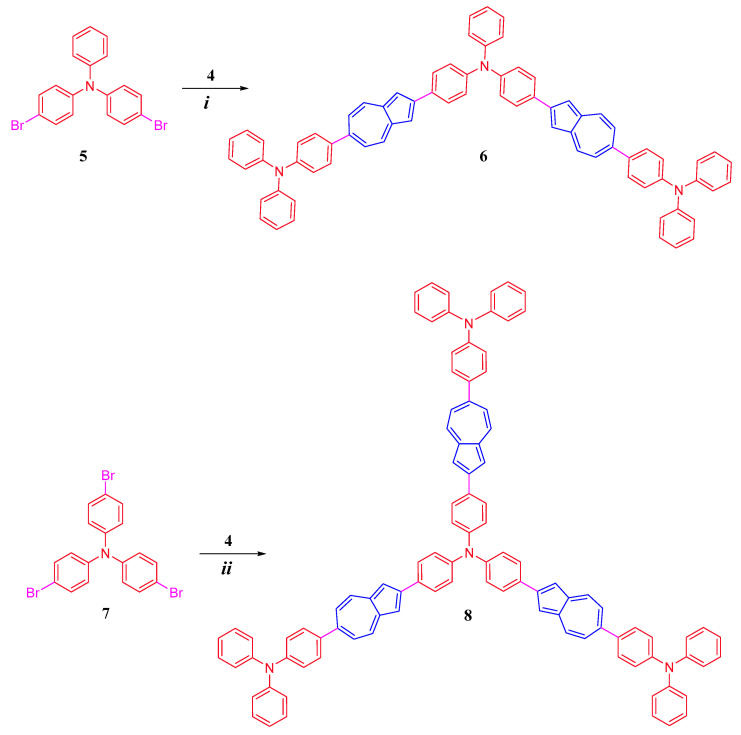
Synthesis of **6** and **8**: (*i*) Pd(PPh_3_)_2_CI_2_, THF: water (4:1), 75–80 °C, 76%; (*ii*) Pd(PPh_3_)_2_CI_2_, THF: water (4:1), 75–80 °C, 72%.

Synthetic pathways leading to conjugated diphenylaniline-azulene co-oligomers of linear and branched structures, 6,6-Bis(N,N-diphenylaniline)-2,2-(4-(diphenylamino)phenyl)-bis-azulene **6** and 6,6,6-tris(N,N-diphenylaniline)-2,2,2-(4-(triphenylamino)-tris-azulene **8**, are shown in Scheme 1 and Scheme 2.

As can be seen from Scheme 1, diborylazulene **2** was obtained by reacting **1** with Bis(pinacolato)diboron in the presence of [IrCl(cod)]_2_, according to the literature procedure [35]. Then, the coupling of amine **3** with borylazulene **2** (1:3 ratio) in the presence of a Pd(PPh_3_)_2_CI_2_ catalyst gives the key compound 2-boryl-6-diphenylaniline-azulene **4** in a high yield of 70%. As can be seen from this scheme, the reaction proceeds regioselectively at the **6** position of the seven-membered azulene ring (Figure S1, Supplementary Materials).

Further, as shown in Scheme 2, the linear co-oligomer bis-azulene **6** was synthesized in a high yield of 76% by reacting dibromotriphenylamine **5** with three equivalents of 2-borylazulene **4** under Suzuki–Miyaura reaction conditions. The expanded tris-azulene **8** co-oligomer was also prepared in a high yield of 72% under similar conditions by reacting tris(4-bromophenyl)amine **7** with four equivalents of 2-borylazulene **4.**

The resulting co-oligomers **6** and **8** are stable brown solids (as opposed to the blue color of the initial azulene). They dissolve well at a temperature of 18–24 °C in solvents such as toluene, chlorobenzene, and methylene chloride.

The structure and purity of the synthesized co-oligomers, 6,6-Bis(N,N-diphenylaniline)-2,2-(4-(diphenylamino)phenyl)-bis-azulene **6** and 6,6,6-tris(N,N-diphenylaniline)-2,2,2-(4-(triphenylamino)-tris-azulene **8**, are determined with spectroscopic methods (Supplementary Materials).

The thermal stability of co-oligomers **6** and **8** was investigated by thermogravimetric analysis (N_2_ medium, heating 10 °C per minute). The onset of degradation **6** and **8** was recorded at 436 and 425 °C, respectively, showing good thermal stability (Figure S7, Supplementary Materials). Differential scanning calorimetry measurements for **6** and **8** were performed at a scan rate of 10 °C per minute. No endothermic or exothermic transitions were observed over the entire scan range (Figure S8, Supplementary Materials).

**Figure 2:**
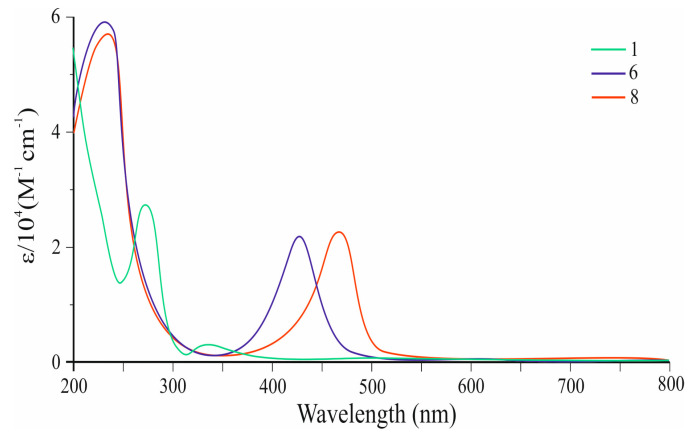
UV–vis spectra of co-oligomers **6** and **8**, as well as azulene **1**.

Figure 2 shows the UV–visible absorption spectra of **6** and **8** (Table 1) in comparison with azulene **1**. It can be seen that co-oligomer **6** exhibits an intense visible absorption band with λ_max_ at 430 nm (ε = 22,447 M^−1^ cm^−1^). The edge of the absorption spectrum **6** is at 475 nm, which corresponds to an optical band gap of 2.61 eV.

Co-oligomer **8** also exhibits an intense visible band λ_max_ at 465 nm and ɛ = 24,621 M^−1^ cm^−1^ (Table 1), which is bathochromically shifted at 35 nm and has a higher intensity than that of **6**. The edge of the absorption spectrum **8** is observed at 490 nm, which corresponds to an optical band gap of 2.53 eV (Figure 2).

**Figure 3:**
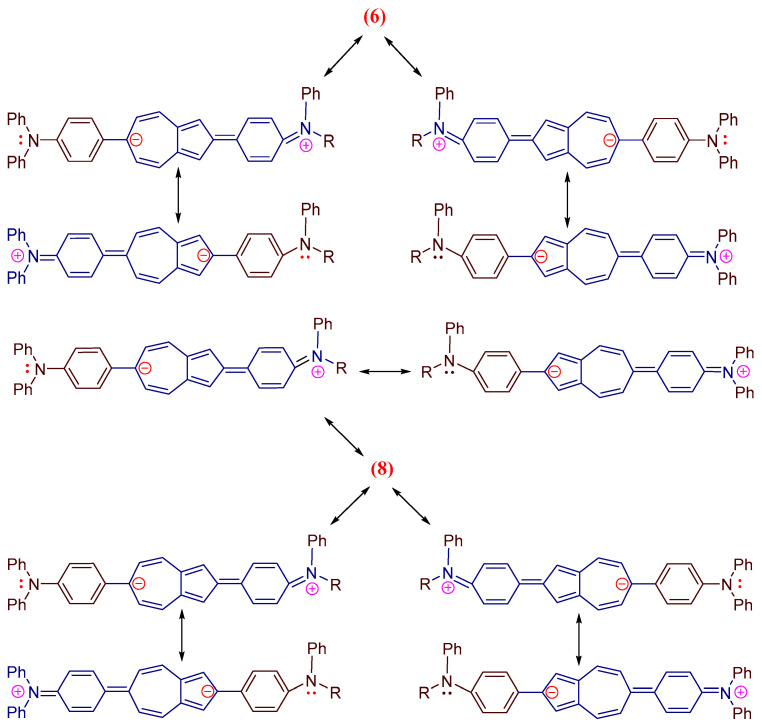
Scheme of resonance structures of co-oligomers **6** and **8**.

This bias is a consequence of the elongation of the π,π-conjugation (Figure 3) and the reduction in the HOMO–LUMO energy gap. It can be seen that the visible electron absorbances of co-oligomers **6** (ε = 22,447 M^−1^ cm^−1^) and **8** (ε = 24,621 M^−1^ cm^−1^) are stronger than those of **1** (ε = 350 M^−1^ cm^−1^) [34].

The electronic absorptions of co-oligomers **6** and **8** in a thin film (Supplementary Materials) were also investigated. As can be seen in Figure S9, the absorption spectra of **6** and **8** in the UV–vis region in spin-coated films (Figure S9a) had a similar appearance to those in the solution (Figure S9b or Figure 2), but the absorptions in the visible region (λmax at 444 and 483 nm, respectively) are shifted by about 14 and 18 nm to the long-wave length region compared to the solution. This red shift may be caused by intermolecular interactions of the compounds in the solid state.

The absorption wavelengths (380–610 nm) of diphenylaniline-azulene co-oligomers **6** and **8** are comparable to those of a number of applied functional materials, such as oligothiophenes and indophenines [34,35,36,37,38,39,40,41,42].

**Table 1:** Electronic absorption and fluorescence values of co-oligomers **6** and **8** (C = 1 × 10^−4^ in DCM).

Co-Oligomers	Absorption Data		Fluorescence Data ^a^	
	Absorption Maximum (nm)	Molar Coefficient (M^−1^ cm^−1^)	Emission Maximum (nm)	QY, % ^b^
6	227	58,826	510	56
	430	22,447		
8	235	57,881	590	65
	465	24,621		

^a^ λ_ex_ (excitation wavelength) 420 nm. ^b^ QY is fluorescence quantum yield and was measured according to the standard procedure; a dilute solution of 1,4-bis- (5-phenyloxazol-2-yl) benzene in ethanol was used as a standard material [42].

**Figure 4:**
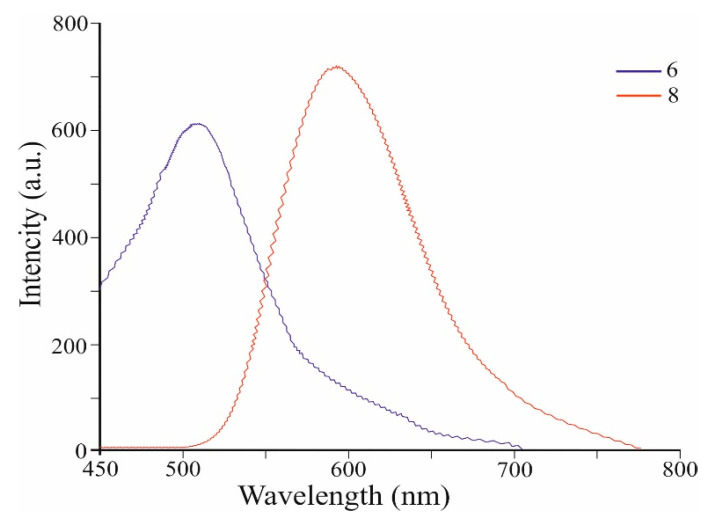
Fluorescence spectra of co-oligomers **6** and **8**.

Figure 4 shows the fluorescence spectra of co-oligomers **6** and **8** (values are given in Table 1). Co-oligomer **6** shows a new intense visible band with a maximum at 510 nm (PLQY of 56%) (when excited at 420 nm) (Table 1). Co-oligomer **8**, when excited also at 420 nm, exhibits intense emission at 590 nm (PLQY of 65%) (Table 1). Figure 4 shows that the emission band of **8** is strongly bathochromically shifted at 80 nm with increasing intensity compared to that of co-oligomer **6** (Table 1). We believe that the significant shift in the fluorescence band **8** is the result of the elongation of the conjugation (Figure 3) and the decrease in the gap between HOMO and LUMO. The resulting ability of co-oligomers **6** and **8** to emit intensively in the green, as well as orange, range is unique due to the absence of such in azulene 1.

**Figure 5:**
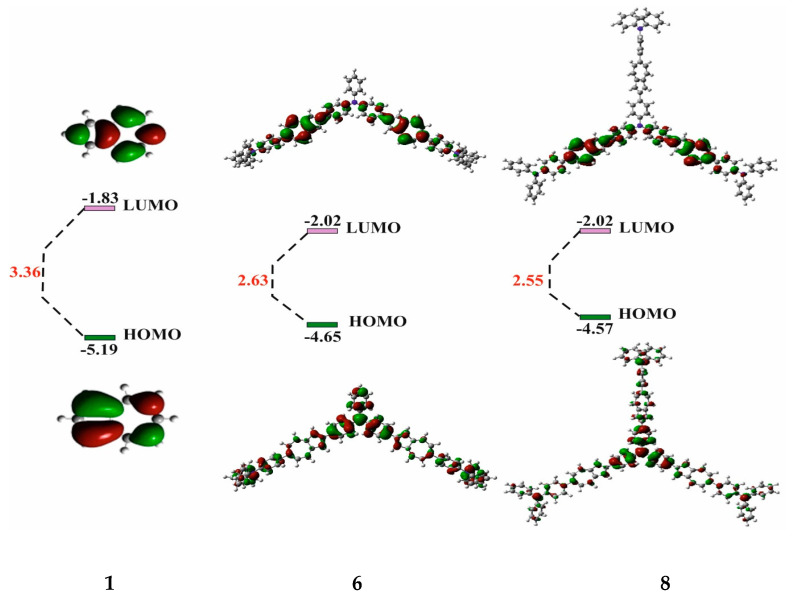
Frontal molecular orbitals of co-oligomers **6** and **8** and initial **1**.

Figure 5 shows the frontal molecular orbitals of co-oligomers **6** and **8** compared to the azulene 1 orbitals obtained by the DFT B3LYP/6- 31G * (Supplementary Materials) method.

It can be seen from this figure that the HOMO orbitals of co-oligomers **6** and **8** are distributed over the skeleton of the entire molecule. This is possible only as a result of the interaction between the HOMO-1 of the azulene cycle and the HOMO of the diphenylaniline fragment [0], since in the HOMO, the C-2 and C-6 atoms of azulene are in the nodal plane, while in the HOMO-1, they have large atomic-orbital coefficients (Figure 1b). It is also shown that in co-oligomers **6** (−4.65 eV) and **8** (−4.57 eV), HOMOs are located higher in level, and LUMOs are lower (−2.02 eV) than the frontal molecular orbitals of the initial azulene **1** (while the gap between HOMO and LUMO decreases by 0.73 eV and 0.81 eV, respectively). This is due to a change in the order of MO levels between initial compound **1** and co-oligomers **6** and **8** [43]. As a result, the prohibited electronic transition of HOMO → LUMO azulene is enabled [43] and, as a result, leads to strong visible absorption and emission. This is what is observed in the electron and fluorescence spectra of co-oligomers **6** and **8** (Figure 2 and Figure 4).

**Figure 6:**
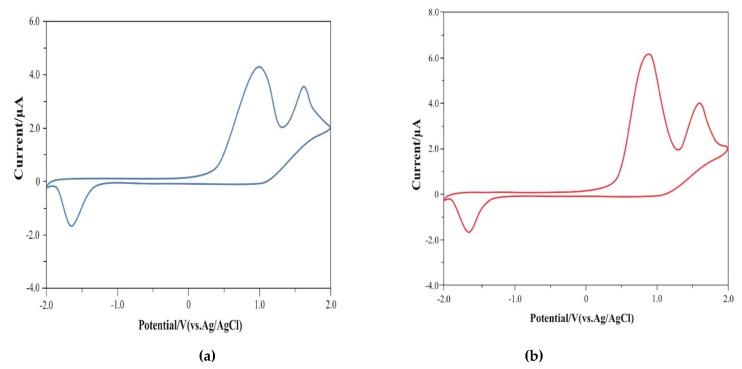
CV of co-oligomers (**a**) **6** and (**b**) **8**.

Figure 6 shows the redox properties of co-oligomers **6** and **8** investigated by cyclic voltammetry (CV) (Supplementary Materials).

As can be seen, co-oligomer **6** exhibits irreversible oxidation waves at 1.0 V and 1.62 V and irreversible reduction at −1.70 V. Co-oligomer **8** also exhibits irreversible oxidation waves at 0.86 V and 1.66 V and irreversible reduction waves at −1.68 V.

In co-oligomer **6**, the onset of oxidation is observed at 0.38 V and reduction at −1.48 V. Based on these data, the energy levels of HOMO and LUMO **6** are −4.82 eV and −2.92 eV, respectively. Similarly, in co-oligomer **8**, the onset of oxidation is observed at 0.39 V and reduction at −1.46 V, which leads to the following levels of HOMO and LUMO: −4.81 eV and −2.94 eV, respectively.

The energy levels of frontal MO were calculated using the formula given in [44].

It is important to note that the HOMO energy levels of co-oligomers **6** and **8** obtained from the experimental electrochemical studies are close to those calculated via the DFT method (Figure 5), while the calculated values of the LUMO levels differ slightly from the experimental ones (the discrepancy is approximately 0.90 V and 0.92 V, respectively).

## 3. Materials and Methods

The following methods were used in the work: For NMR spectrometry, a JNM-ECA 500 spectrometer (Jeol, Tokyo, Japan) (operating frequencies 500 MHz for ^1^Н and 126 MHz for ^13^C; solvent СDCl_3_, internal standard TMS) was used; for FT-IR spectrometry, an Avatar-360 in KBr (Thermo Nicolet, Waltham, MA, USA) was used; for HRMS spectrometer, UV–vis spectra **6** and **8** were Thermo Electron Corporation DFS (Thermo Fisher Scientific Inc., Waltham, MA, USA), element analyzer CHNS-O UNICUBE (Elementar Analysensysteme, Frankfurt, Germany), and Melting Point M-560 (Buchi, Luzern, Switzerland) for m.p.

Shimadzu UV-1800 spectrophotometer (Shimadzu, Kyoto, Japan); Agilent Cary Eclipse spectrofluorometer (Agilent Technologies, Palo Alto, CA, USA); PalmSens analyzer (PalmSens, Enschede, The Netherlands) for cyclic voltammetry (CV); TGA Q500 device (in N_2_ current; heating at 10° per minute; range 20–500 °C) for thermogravimetric studies (TA Instruments, Newark, DE, USA); DSC Q2000 instrument (in current N_2_; heating at 5° per minute; interval 20–300 °C) for differential scanning calorimetry (TA Instruments, USA). The following materials (i.e., marketed reagents and solvents) were used: precursors **1**, **3**, **5**, **7**, (Bpin)_2_, cyclooctadiene iridium chloride dimension, bis (triphenylphosphine) palladium chloride, 2,2′-bpy, tetrahydrofuran, dichloromethane, and others.

Precursor 2,6-Diborilazulene **2** was prepared according to the method [36].

### 3.1. 6-(N,N-diphenylaniline)-2-(tetramethyl-borolanyl)-azulene (***4***)

To the mixture of 140 mg (0.43 mmol) of **3** and 491 mg (1.30 mmol) of **2** in 10 mL of degassed THF/H_2_O (4:1 ratio) under an argon atmosphere was added 16 mg (0.02 mmol) of Pd(PPh_3_)_2_CI_2_ and 181 mg (1.30 mmol) of potassium carbonate. Then, it was boiled for 8 h at 75–80 °C. The mixture was cooled and extracted with DCM (3 × 18 mL). Then, it was dried with magnesium sulfate, and DCM was removed on a rotary evaporator (Buchi, Switzerland). The resulting substance was purified via chromatography (SiO_2_ column, C_6_H_12_/DCM mixture, 9:1 ratio) and recrystallized from DCM. A total of 348 mg of dark green solid (70% yield) was obtained. M.p. 104–105.5 °C. HRMS (ESI) *m*/*z*: C_34_H_33_BN0_2_ [M + H]^+^: 498.0384, found 498.0359. Anal. calcd for C_34_H_32_BN0_2_; C 82.08, H 6.48, N 2.82. Found: C 81.89, H 6.34, N 2.89. IR (ν, cm^–1^): 2935, 2862, 1576, 1426, 1412, 1330, 1232, 1160, 1058, 690. ^1^H NMR (Figure S1. Supplementary Materials). ^13^C NMR (Figure S2. Supplementary Materials).

### 3.2. 6,6-Bis(N,N-diphenylaniline)-2,2-(4-(diphenylamino)phenyl)-bis-azulene (***6***)

To the mixture of 173 mg (0.43 mmol) of **5** and 646 mg (1.30 mmol) of **4** in 12 mL of degassed THF/H_2_O (4:1 ratio) under an argon atmosphere, 16 mg (0.02 mmol) of Pd(PPh_3_)_2_CI_2_ and 181 mg (1.30 mmol) of potassium carbonate were added. Then, it was boiled for 8 h at 75–80 °C. Then, it was cooled and extracted with DCM (3 × 18 mL), dried with magnesium sulfate, and the DCM was removed on a rotary evaporator (Buchi, Switzerland). The resulting substance was purified by chromatography (SiO_2_ column, mixture C_6_H_12_/DCM, ratio 4:1) to obtain 746 mg of brown powder (yield 76%). HRMS (ESI) *m*/*z*: C_74_H_54_N_3_ [M + H]^+^: 984.0541, found 984.0517. Anal. calcd for C_74_H_53_N_3_; C 90.30, H 5.43, N 4.27. Found C 90.10, H 5.29, N 4.34. IR (ν, cm^–1^): 2919, 2860, 1579, 1452, 1409, 1331, 1268, 1170, 1070, 691. ^1^H NMR (Figure S3. Supplementary Materials). ^13^C NMR (Figure S4. Supplementary Materials).

### 3.3. 6,6,6-tris(N,N-diphenylaniline)-2,2,2-(4-(triphenylamino)-tris-azulene (***8***)

To the mixture of 207 mg (0.43 mmol) of **7** and 940 mg (1.93 mmol) of **4** in 12 mL of degassed THF/H_2_O (4:1 ratio) under an argon atmosphere, 16 mg (0.02 mmol) of Pd(PPh_3_)_2_CI_2_ and 270 mg (1.93 mmol) of potassium carbonate were added. Then, it was boiled for 8 h at 75–80 °C. Then, it was cooled, and extracted with DCM (3 × 18 mL), dried with magnesium sulfate, and the DCM was removed on a rotary evaporator (Buchi, Switzerland). The obtained substance was purified by chromatography (SiO_2_ column, mixture C_6_H_12_/DCM, ratio 4:1) to obtain 973 mg of brown powder (yield 72%). HRMS (ESI) *m*/*z*: C_102_H_73_N_4_ [M + H]^+^: 1353.0642, found 1353.0619. Anal. calcd for C_102_H_72_N_4_; C90.50, H5.36, N4.14. Found C90.30, H5.23, N4.21. IR (ν, cm^–1^): 2924, 2855, 1587, 1488, 1415, 1325, 1275, 1173, 1072, 694. ^1^H NMR (Figure S5. Supplementary Materials). ^13^C NMR (Figure S6. Supplementary Materials).

## 4. Conclusions

In this work, for the first time, conjugated diphenylaniline-azulene co-oligomers of linear and branched structure were synthesized: 6,6-bis (N, Ndiphenylaniline)-2,2-(4-(diphenylamino) phenyl)-bis-azulene **6** and 6,6,6-tris (N, Ndiphenylaniline)-2,2,2-(4- (triphenylamino)-tris-azulene **8**. The obtained co-oligomers have a pronounced ability to absorb and emit visible light in the range of 400–700 nm.

The obtained co-oligomers possess good solubility and thermodynamic stability and have a higher HOMO compared to oligothiophenes.

The results provide a good opportunity to design new diphenylaniline-azulene-based co-oligomers for electronic and photonic applications.

## Data Availability

The data presented in the study are included in the article/Supplementary Material, further inquiries can be directed to the corresponding authors.

## Supplementary Materials

The following supporting information can be downloaded at: https://www.mdpi.com/article/10.3390/molecules29215041/s1, Figure S1: ^1^H NMR spectra of 6-(N,N-diphenylaniline)-2-(4,4,5,5-tetramethyl-1,3,2-dioxaborolanyl)-azulene 4; Figure S2: ^13^C NMR spectra of 6-(N,N-diphenylaniline)-2-(4,4,5,5-tetramethyl-1,3,2-dioxaborolanyl)-azulene 4; Figure S3: ^1^H NMR spectra of 6,6-Bis(N,N-diphenylaniline)- 2,2-(4-(diphenylamino)phenyl)-bis-azulene 6; Figure S4: ^13^C NMR spectra of 6,6-Bis(N,N-diphenylaniline)- 2,2-(4-(diphenylamino)phenyl)-bis-azulene 6; Figure S5: ^1^H NMR spectra of 6,6,6-tris(N,N-diphenylaniline)-2,2,2-(4-(triphenylamino)-tris-azulene 8; Figure S6: ^13^C NMR spectra of 6,6,6-tris(N,N-diphenylaniline)-2,2,2-(4-(triphenylamino)-tris-azulene 8; Figure S7: Thermogravimetric measurements of co-oligomers 6 and 8; Figure S8: Differential scanning calorimetry measurements of co-oligomers 6 (a) and 8 (b); Figure S9: UV–vis absorption spectra of 6 and 8. Density Functional Theory (DFT) Calculations; Table S1: Atomic coordinates of optimized geometry of 6; Table S2: Atomic coordinates of optimized geometry of 8; Cyclic voltammetry studies.
