# Supplementary material for: Conjugated Diphenylaniline-Azulene Co-Oligomers with Intense Absorption and Emission in the Visible Region
Source: Molecules. 2024 Oct 25;29(21):5041. doi: 10.3390/molecules29215041 (PMC11547525; doi:10.3390/molecules29215041)
Supplement: Supplementary file 1 [file molecules-29-05041-s001.zip › molecules-3268685-supplementary.pdf]

Supplementary Materials

Contents

1. Spectra of compounds.

2. Thermal properties.

3. Preparation of thin films and their absorption spectra.

4. Density functional theory (DFT) calculations.

5. Cyclic voltammetry studies.

1. Spectra of compounds

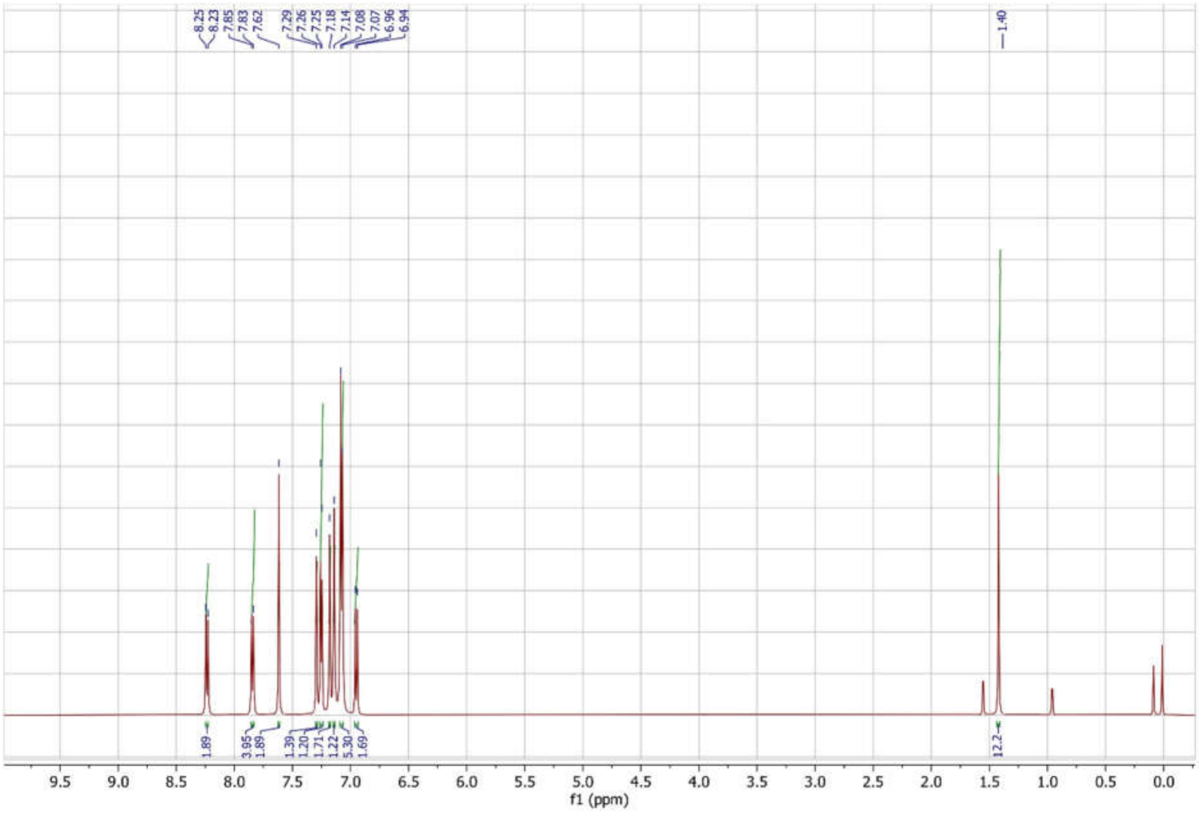

Figure S1. ^{1}H NMR spectra of 6-(N,N-diphenylaniline)-2-(4,4,5,5-tetramethyl-1,3,2-dioxaborolanyl)-azulene **4**.

^{1}H NMR: δ 8.25 (d, J = 9.40 Hz, 2H), 7.84 (d, J = 8.3 Hz, 4H), 7.62 (s, 2H), 7.29 – 6.94 (m, 12H), 1.40 (s, 12H).

In this spectrum, the proton signals at C-1 and C-3 (singlet at 7.62 δ) are consistent with the signals of the same protons of the initial molecule **2** [1], which confirms the structure of molecule **4**. In the case of substitution of the azulene position **2** with a diphenylaniline group, a significant

shift of proton signals during C-1 and C-3 in a strong field would occur by an average of 1.0 δ due to an increase in electron density in the five-membered ring [2].

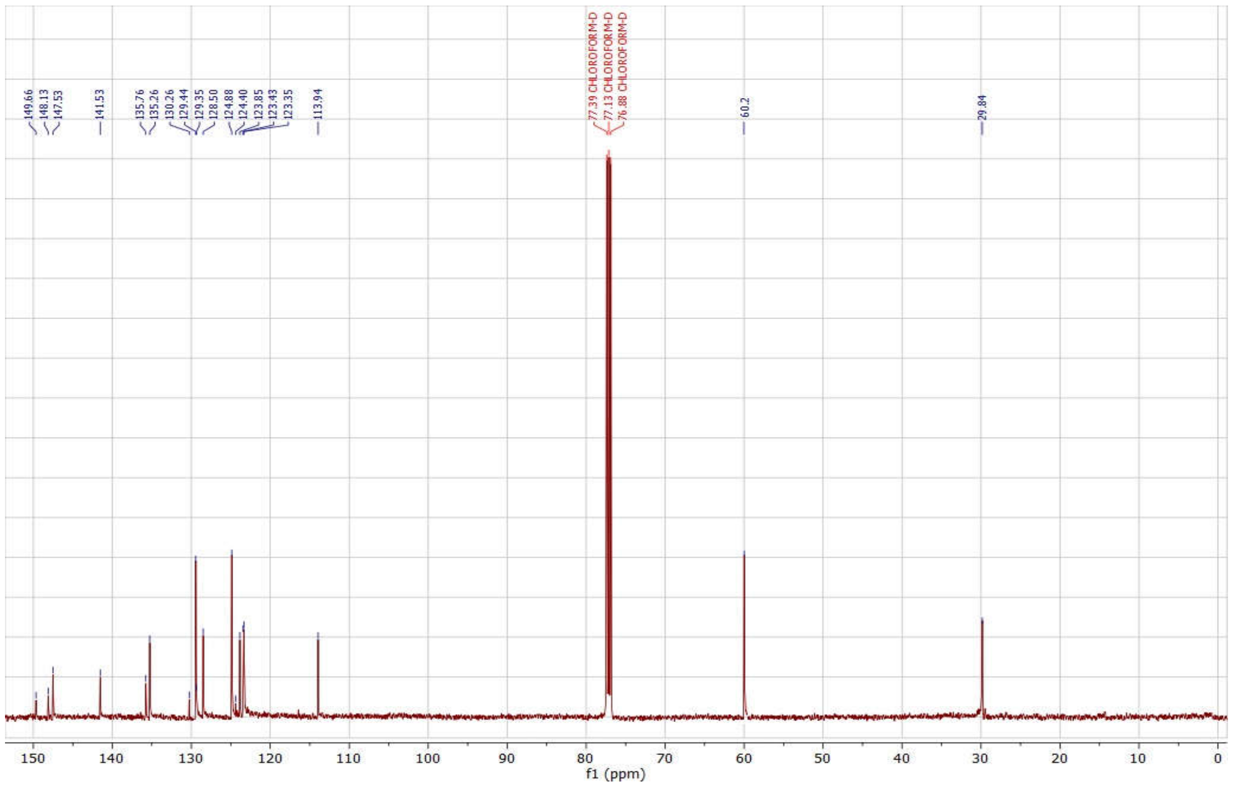

Figure S2. ^{13}C NMR spectra of 6-(N,N-diphenylaniline)-2-(4,4,5,5-tetramethyl-1,3,2-dioxaborolanyl)-azulene **4**.

^{13}C NMR: δ 149.66, 148.13, 147.54, 141.53, 135.77, 135.26, 130.26, 129.44, 129.35, 128.50, 124.88, 124.40, 123.85, 123.43, 123.35, 113.94, 60.20, 29.84.

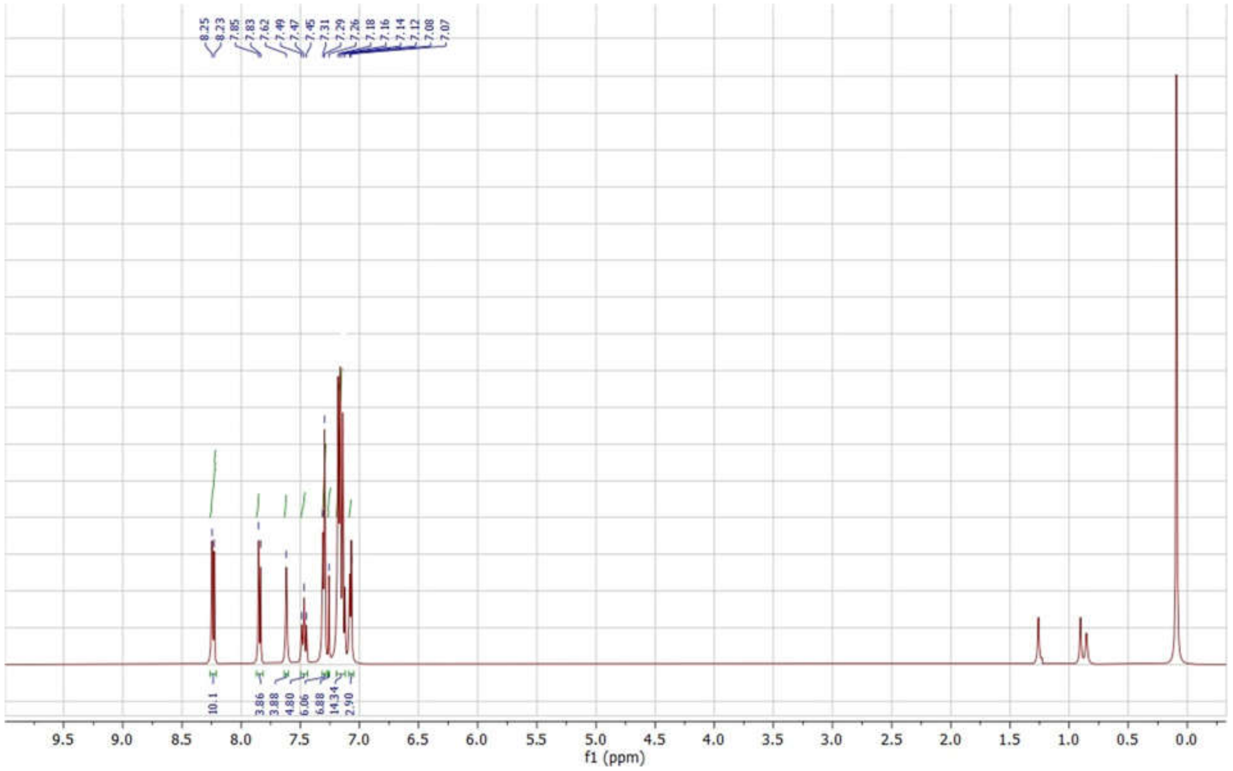

Figure S3. ^{1}H NMR spectra of 6,6-Bis(N,N-diphenylaniline)-2,2-(4-(diphenylamino)phenyl)-bis-azulene **6**.

^{1}H NMR: δ 8.24 (d, J = 9.4 Hz, 10H), 7.88 (d, J = 12.3 Hz, 4H), 7.62 (s, 4H), 7.47 (t, J = 9.9 Hz, 5H),7.37 – 7.04 (m, 30H).

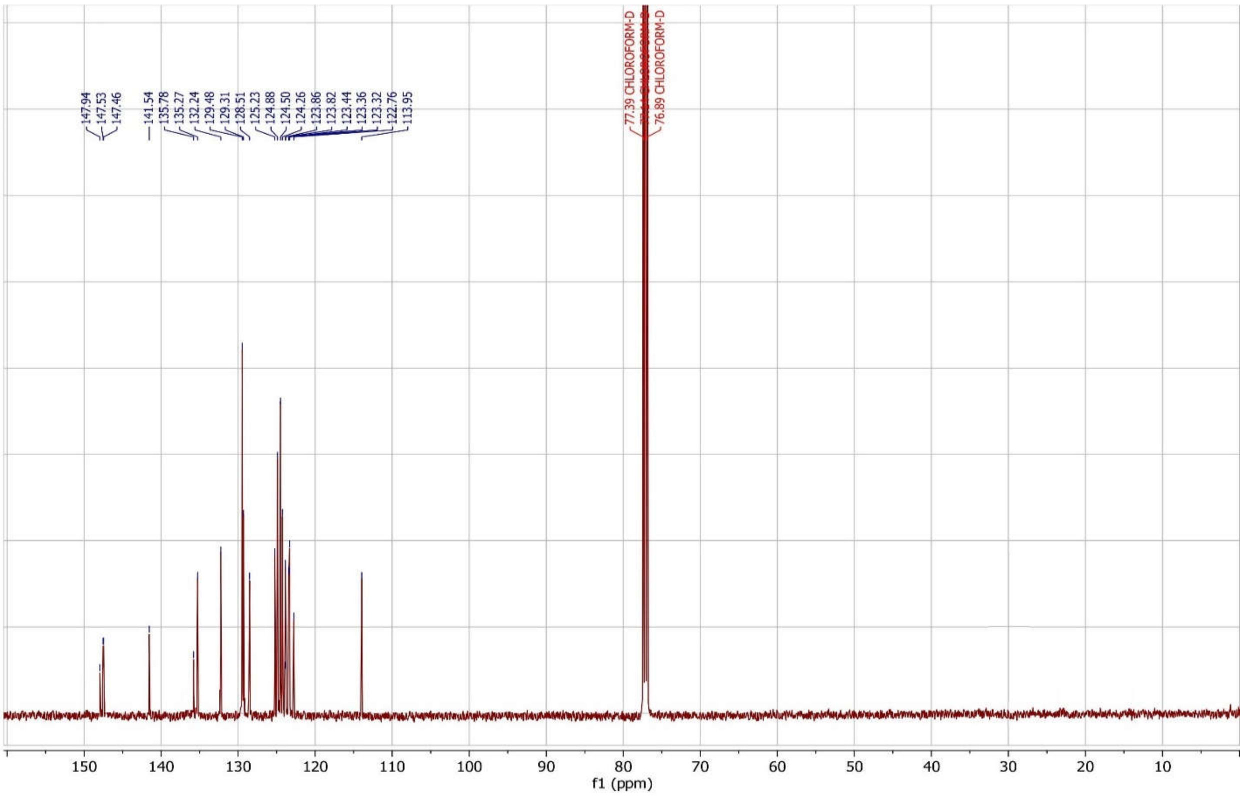

Figure S4. ^{13}C NMR spectra of 6,6-Bis(N,N-diphenylaniline)- 2,2-(4-(diphenylamino)phenyl)-bis-azulene **6**.

^{13}C NMR: δ 147.84, 147.63, 147.44, 141.64, 135.88, 135.37, 132.44, 129.78, 129.21, 128.41, 125.33, 124.78, 124.47, 124.36, 123.76, 123.72, 123.34, 123.46, 123.22, 122.61, 113.84

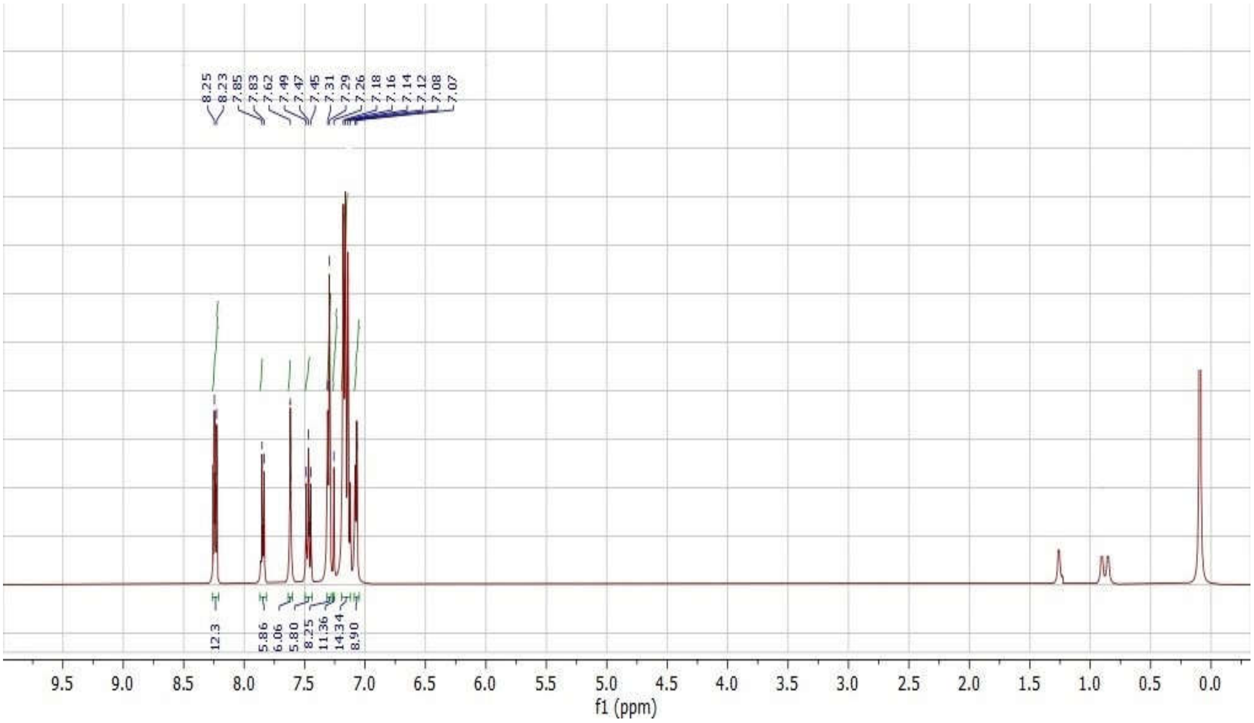

Figure S5. ^{1}H NMR spectra of 6,6,6-tris(N,N-diphenylaniline)-2,2,2-(4-(triphenylamino)-tris-azulene **8**.

^{1}H NMR: δ 8.24 (d, J = 9.4 Hz, 12H), 7.88 (d, J = 12.3 Hz, 6H), 7.62 (s, 6H), 7.47 (t, J = 9.9 Hz, 6H),7.37 – 7.04 (m, 42H).

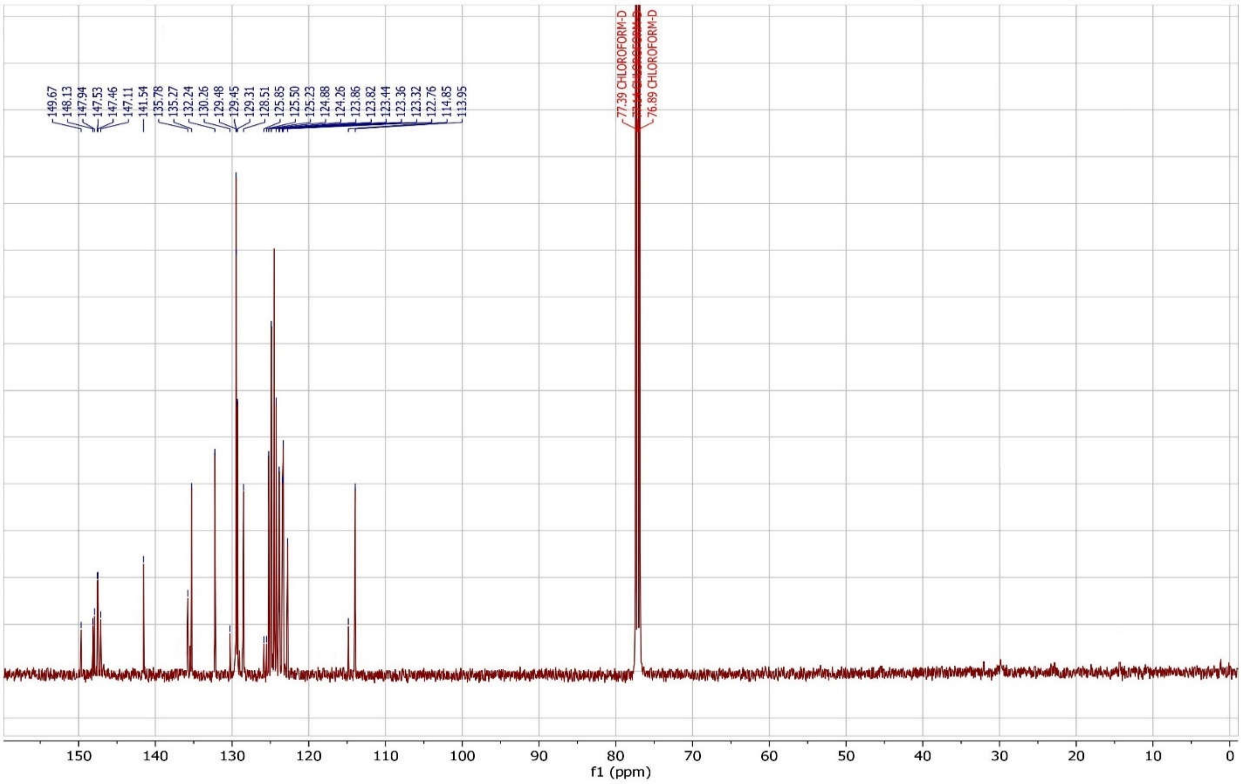

Figure S6. ^{13}C NMR spectra of 6,6,6-tris(N,N-diphenylaniline)-2,2,2-(4-(triphenylamino)-tris-azulene **8**.

^{13}C NMR: δ 149.57, 148.23, 147.84, 147.43, 147.66, 147.21, 141.44, 135.68, 137.37, 132.14, 130.36, 129.58, 129.55, 129.21, 128.41, 125.75, 125.46, 125.32, 124.77, 124.46, 123.36, 13.52, 123.14, 123.66, 123.13, 122.36, 114.55, 113.25.

2. Thermal properties

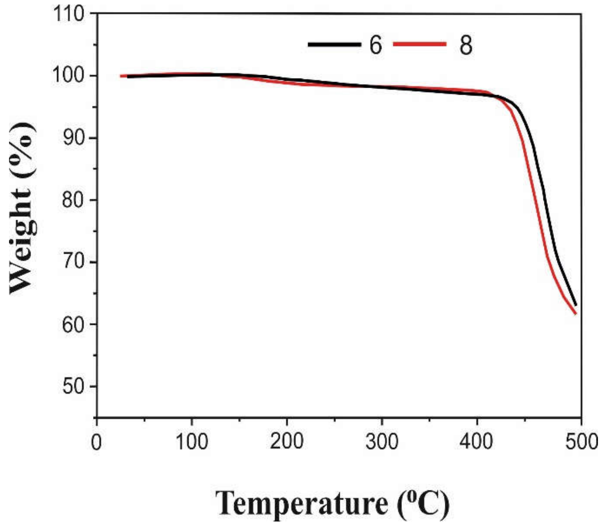

Figure S7. Thermogravimetric measurements of co-oligomers **6** and **8.**

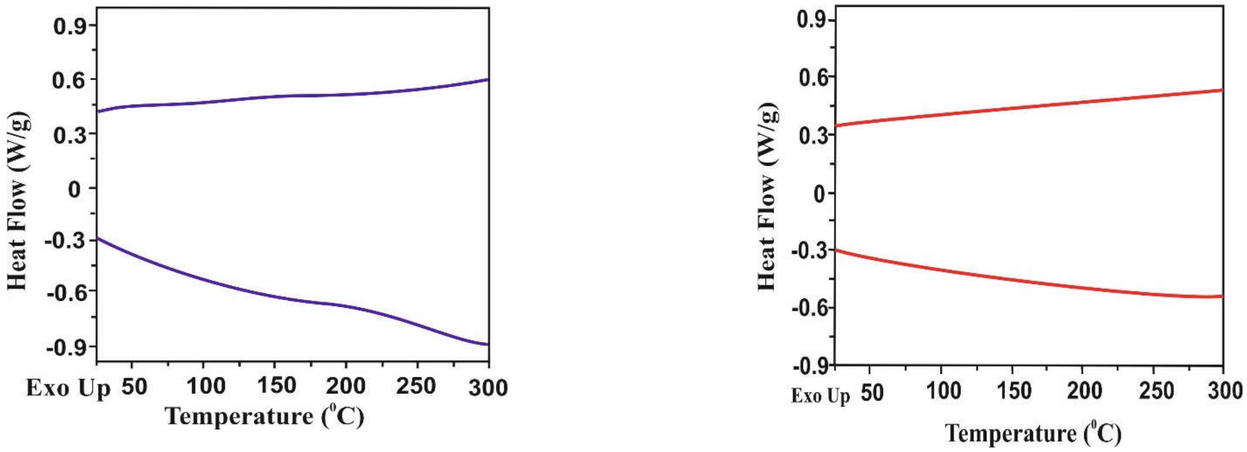

(a) (b)

Figure S8. Differential scanning calorimetry measurements of co-oligomers **6** (a) and **8** (b).

3. Preparation of thin films and their absorption spectra

Thin films of co-oligomers **6** and **8** were prepared by spin-coating a solution of each compound (10 mg/ml in chlorobenzene) at 1000 rpm for 60 seconds.

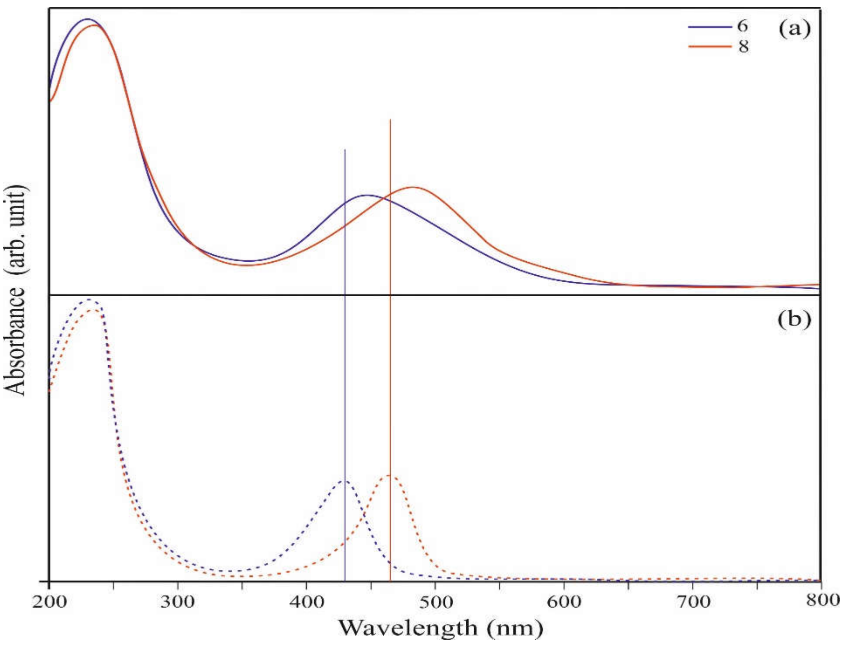

Figure S9. UV–Vis absorption spectra of **6** and **8**: (a) in spin coated film; (b) in DCM.

4. Density Functional Theory (DFT) Calculations

To characterize the geometry and orbitals of the HOMO–LUMO of co-oligomers **6** and **8**, DFT calculations based on the level of B3LYP/6-31G * (d, p) Gaussian 16 were used. The geometry of the molecules was optimized in a singlet state using the keywords Opt and Freq, and the results were recorded in a .chk file to visualize the boundary orbitals.

After the correct completion of the calculations, the .chk file was opened in GaussView 6.0, and the HOMO–LUMO orbitals were visualized from it using the built-in GaussView tool. Then, we obtained the energies of the boundary orbitals in Hartree units and recalculated them into eV, multiplying by the recalculation coefficient 27.2114.

Table S1. Atomic coordinates of optimized geometry of **6.**

Center Number	Atomic Number	Atomic Type	Coordinates (Angstroms) X	Y	Z
1	7	0	0.000016	5.209712	-0.000009
2	6	0	0.000032	6.641096	0.000008
3	6	0	-0.882430	7.353068	0.833264
4	6	0	-0.884358	8.750167	0.824065
5	6	0	0.000046	9.457247	0.000018
6	6	0	0.884443	8.750164	-0.824035
7	6	0	0.882501	7.353067	-0.833245
8	6	0	-1.236834	4.500898	-0.003050
9	6	0	-2.324929	4.956612	-0.772650
10	6	0	-3.534011	4.267328	-0.765797
11	6	0	-3.711757	3.090761	-0.005472
12	6	0	-2.610105	2.643332	0.756374
13	6	0	-1.400737	3.332112	0.765894
14	6	0	1.236836	4.500851	0.003087
15	6	0	2.324975	4.956620	0.772590
16	6	0	3.534029	4.267285	0.765791
17	6	0	3.711710	3.090617	0.005611
18	6	0	2.610005	2.643124	-0.756128
19	6	0	1.400663	3.331943	-0.765694
20	6	0	-9.616429	1.091014	-0.447769
21	6	0	-9.586557	-0.252657	-0.009380
22	6	0	-8.447079	-0.965039	0.429476
23	6	0	-7.118814	-0.553799	0.553948
24	6	0	-6.534148	0.682198	0.265004
25	6	0	-7.223288	1.893795	-0.280723
26	6	0	-8.584204	2.022735	-0.570988
27	6	0	-5.179407	1.022003	0.415473
28	6	0	-4.984971	2.365768	-0.006488
29	6	0	-6.239153	2.885059	-0.429519
30	6	0	8.446998	-0.965231	-0.429291
31	6	0	9.586482	-0.252830	0.009510
32	6	0	9.616369	1.090840	0.447894
33	6	0	8.584149	2.022569	0.571104
34	6	0	7.223229	1.893625	0.280862
35	6	0	6.534065	0.681997	-0.264777
36	6	0	7.118723	-0.554005	-0.553713
37	6	0	6.239108	2.884907	0.429608
38	6	0	4.984906	2.365597	0.006654
39	6	0	5.179323	1.021803	-0.415227
40	7	0	-14.599922	-3.099852	-0.009067
41	6	0	-15.309485	-3.309380	1.216062
42	6	0	-15.896771	-4.557781	1.492447
43	6	0	-16.600060	-4.755825	2.683379
44	6	0	-16.715379	-3.722791	3.621671
45	6	0	-16.125483	-2.481760	3.351606
46	6	0	-15.434747	-2.269421	2.155833
47	6	0	-15.144877	-3.600890	-1.233953
48	6	0	-16.517444	-3.461701	-1.510446
49	6	0	-17.049280	-3.963205	-2.701159
50	6	0	-16.222977	-4.594344	-3.639108

51	6	0	-14.855355	-4.727438	-3.368959
52	6	0	-14.317742	-4.244037	-2.173385
53	6	0	-13.361652	-2.397393	-0.009534
54	6	0	-12.439638	-2.559301	1.042909
55	6	0	-11.230979	-1.866232	1.039872
56	6	0	-10.884119	-0.990540	-0.009754
57	6	0	-11.813796	-0.840234	-1.059493
58	6	0	-13.028250	-1.523086	-1.062336
59	7	0	14.599961	-3.099849	0.008948
60	6	0	15.144902	-3.601030	1.233776
61	6	0	16.517461	-3.461854	1.510324
62	6	0	17.049273	-3.963489	2.700991
63	6	0	16.222958	-4.594749	3.638848
64	6	0	14.855345	-4.727830	3.368647
65	6	0	14.317755	-4.244299	2.173117
66	6	0	13.361644	-2.397454	0.009492
67	6	0	13.028230	-1.523228	1.062354
68	6	0	11.813749	-0.840421	1.059569
69	6	0	10.884063	-0.990705	0.009840
70	6	0	11.230922	-1.866339	-1.039836
71	6	0	12.439612	-2.559359	-1.042933
72	6	0	15.309568	-3.309121	-1.216195
73	6	0	15.897001	-4.557416	-1.492755
74	6	0	16.600325	-4.755208	-2.683707
75	6	0	16.715538	-3.722027	-3.621851
76	6	0	16.125499	-2.481102	-3.351612
77	6	0	15.434725	-2.269015	-2.155815
78	1	0	-1.558877	6.808453	1.482219
79	1	0	-1.570103	9.286373	1.472656
80	1	0	0.000051	10.542240	0.000022
81	1	0	1.570191	9.286369	-1.472623
82	1	0	1.558941	6.808449	-1.482205
83	1	0	-2.212149	5.846561	-1.381175
84	1	0	-4.346430	4.628558	-1.387138
85	1	0	-2.714678	1.760277	1.377551
86	1	0	-0.579043	2.975530	1.376371
87	1	0	2.212252	5.846642	1.381017
88	1	0	4.346472	4.628558	1.387074
89	1	0	2.714513	1.759961	-1.377162
90	1	0	0.578928	2.975296	-1.376078
91	1	0	-10.601521	1.466217	-0.709975
92	1	0	-8.628065	-2.003656	0.691161
93	1	0	-6.432634	-1.314822	0.924418
94	1	0	-8.887486	3.001577	-0.941323
95	1	0	-4.408916	0.347499	0.763367
96	1	0	-6.425284	3.892189	-0.776931
97	1	0	8.627984	-2.003848	-0.690969
98	1	0	10.601466	1.466040	0.710082
99	1	0	8.887439	3.001418	0.941412
100	1	0	6.432530	-1.315043	-0.924128
101	1	0	6.425253	3.892053	0.776963
102	1	0	4.408816	0.347273	-0.763037
103	1	0	-15.798369	-5.363743	0.774066
104	1	0	-17.047611	-5.724448	2.883113
105	1	0	-17.256704	-3.882066	4.548339
106	1	0	-16.214048	-1.670156	4.067059
107	1	0	-14.992295	-1.302619	1.943810
108	1	0	-17.157673	-2.961999	-0.792294
109	1	0	-18.110075	-3.848300	-2.901001
110	1	0	-16.638176	-4.976865	-4.565610
111	1	0	-14.205176	-5.221554	-4.084174
112	1	0	-13.261144	-4.362409	-1.961184
113	1	0	-12.677840	-3.224544	1.864645

114	1	0	-10.551349	-1.993494	1.876054
115	1	0	-11.574988	-0.191425	-1.895739
116	1	0	-13.721661	-1.386854	-1.884016
117	1	0	17.157703	-2.962055	0.792251
118	1	0	18.110062	-3.848591	2.900872
119	1	0	16.638140	-4.977373	4.565314
120	1	0	14.205154	-5.222036	4.083790
121	1	0	13.261165	-4.362667	1.960874
122	1	0	13.721643	-1.387028	1.884036
123	1	0	11.574926	-0.191675	1.895861
124	1	0	10.551267	-1.993603	-1.875996
125	1	0	12.677820	-3.224565	-1.864695
126	1	0	15.798687	-5.363493	-0.774491
127	1	0	17.047988	-5.723753	-2.883573
128	1	0	17.256888	-3.881108	-4.548537
129	1	0	16.213981	-1.669382	-4.066944
130	1	0	14.992166	-1.302291	-1.943659

Method: DFT B3LYP 6-31G*
Key word: opt freq
E_{total} (RB3LYP)= -3015.311973 Hartree

Table S2. Atomic coordinates of optimized geometry of **8.**

Center Number	Atomic Number	Atomic Type	Coordinates (Angstroms) X	Y	Z
1	7	0	0.002269	0.000027	-0.001192
2	6	0	0.717952	-1.234013	0.008182
3	6	0	1.877505	-1.387611	0.792488
4	6	0	2.575416	-2.591804	0.793479
5	6	0	2.146002	-3.697250	0.026744
6	6	0	0.978294	-3.529356	-0.749486
7	6	0	0.280115	-2.325456	-0.766622
8	6	0	0.712706	1.237070	-0.010418
9	6	0	1.872075	1.395382	-0.794068
10	6	0	2.564936	2.602485	-0.794881
11	6	0	2.130404	3.706282	-0.028663
12	6	0	0.962928	3.533670	0.746880
13	6	0	0.269826	2.326838	0.763891
14	6	0	-1.424747	-0.003054	-0.001176
15	6	0	-2.142285	-0.917695	0.793381
16	6	0	-3.534202	-0.920821	0.785120
17	6	0	-4.272405	-0.009162	-0.001006
18	6	0	-3.538214	0.905632	-0.787246
19	6	0	-2.146296	0.908474	-0.795684
20	6	0	6.194875	8.427721	-0.528145
21	6	0	5.496742	9.568383	-0.068989
22	6	0	4.162313	9.601228	0.397375
23	6	0	3.224071	8.574989	0.520222
24	6	0	3.342205	7.214319	0.224033
25	6	0	4.547706	6.518290	-0.325874
26	6	0	5.780656	7.099007	-0.635290
27	6	0	2.348755	6.234277	0.385313
28	6	0	2.859990	4.976894	-0.037807
29	6	0	4.201076	5.164684	-0.471043
30	6	0	-10.395986	0.657777	-1.062833
31	6	0	-11.032742	-0.023227	-0.000094
32	6	0	-10.392856	-0.701653	1.062434
33	6	0	-9.034577	-0.870474	1.336768
34	6	0	-7.916357	-0.420349	0.629594

35	6	0	-7.918207	0.386727	-0.630747
36	6	0	-9.038489	0.832184	-1.337604
37	6	0	-6.570409	-0.639433	0.966518
38	6	0	-5.738205	-0.012261	-0.000863
39	6	0	-6.573260	0.611392	-0.968044
40	7	0	8.367151	14.566519	-0.099922
41	6	0	7.709878	15.778139	0.285492
42	6	0	7.904485	16.958322	-0.455421
43	6	0	7.272282	18.143057	-0.069579
44	6	0	6.426976	18.167302	1.046543
45	6	0	6.226626	16.992037	1.781200
46	6	0	6.867735	15.806638	1.412549
47	6	0	9.742939	14.605905	-0.492714
48	6	0	10.668153	15.374639	0.237126
49	6	0	12.008062	15.422113	-0.155998
50	6	0	12.449116	14.694849	-1.268390
51	6	0	11.531079	13.923636	-1.991960
52	6	0	10.186020	13.883395	-1.616062
53	6	0	7.658687	13.332778	-0.092488
54	6	0	6.303465	13.276149	-0.472932
55	6	0	5.611679	12.067327	-0.454586
56	6	0	6.239622	10.861741	-0.077298
57	6	0	7.599388	10.930406	0.292034
58	6	0	8.294870	12.137246	0.295537
59	7	0	-16.798058	-0.034846	0.000930
60	6	0	-17.520768	0.832365	0.880727
61	6	0	-18.645217	0.357541	1.580666
62	6	0	-19.356950	1.209615	2.428922
63	6	0	-18.952121	2.538450	2.605443
64	6	0	-17.829007	3.010921	1.915181
65	6	0	-17.121164	2.170782	1.051909
66	6	0	-15.374493	-0.031967	0.000686
67	6	0	-14.652850	0.126335	1.199927
68	6	0	-13.259626	0.132074	1.193794
69	6	0	-12.525374	-0.026234	0.000177
70	6	0	-13.259413	-0.187487	-1.193173
71	6	0	-14.652650	-0.187356	-1.198812
72	6	0	-17.517504	-0.904998	-0.878653
73	6	0	-18.643933	-0.434697	-1.578456
74	6	0	-19.352403	-1.289662	-2.426538
75	6	0	-18.942326	-2.616896	-2.603001
76	6	0	-17.817255	-3.084855	-1.912854
77	6	0	-17.112630	-2.241837	-1.049755
78	6	0	6.229368	-8.402061	0.529137
79	6	0	5.536730	-9.545299	0.068047
80	6	0	4.203210	-9.583333	-0.400535
81	6	0	3.260882	-8.560933	-0.524078
82	6	0	3.372848	-7.200012	-0.226635
83	6	0	4.574605	-6.499350	0.325552
84	6	0	5.809476	-7.075150	0.636497
85	6	0	2.375555	-6.224015	-0.388703
86	6	0	2.880895	-4.964802	0.036063
87	6	0	4.222119	-5.147305	0.471141
88	7	0	8.427813	-14.531501	0.100932
89	6	0	9.803040	-14.565438	0.496188
90	6	0	10.732765	-15.329790	-0.232517
91	6	0	12.072144	-15.371973	0.163012
92	6	0	12.508140	-14.643698	1.276737
93	6	0	11.585589	-13.876833	1.999186
94	6	0	10.241065	-13.841901	1.620856
95	6	0	7.776281	-15.745651	-0.286323
96	6	0	7.974067	-16.925277	0.454624
97	6	0	7.347502	-18.112433	0.067022

98	6	0	6.504714	-18.139709	-1.050934
99	6	0	6.301186	-16.965016	-1.785635
100	6	0	6.936696	-15.777158	-1.415202
101	6	0	7.714234	-13.300738	0.092925
102	6	0	8.346202	-12.102274	-0.292957
103	6	0	7.645697	-10.898346	-0.289972
104	6	0	6.284933	-10.835581	0.076743
105	6	0	5.661275	-12.044057	0.451898
106	6	0	6.358025	-13.250014	0.470697
107	1	0	2.219633	-0.562090	1.406112
108	1	0	3.451111	-2.689433	1.426051
109	1	0	0.631862	-4.344951	-1.375015
110	1	0	-0.602373	-2.219239	-1.387124
111	1	0	2.218023	0.571183	-1.407327
112	1	0	3.440593	2.703679	-1.426951
113	1	0	0.612713	4.347958	1.372010
114	1	0	-0.612570	2.217049	1.383901
115	1	0	-1.602387	-1.624424	1.413136
116	1	0	-4.059387	-1.646955	1.395969
117	1	0	-4.066592	1.629534	-1.397995
118	1	0	-1.609510	1.617533	-1.415482
119	1	0	7.204527	8.618873	-0.880103
120	1	0	3.820559	10.573109	0.741550
121	1	0	2.257660	8.878677	0.921530
122	1	0	6.527665	6.413347	-1.034146
123	1	0	1.345060	6.425300	0.739950
124	1	0	4.869824	4.390429	-0.821715
125	1	0	-11.067979	1.146668	-1.762356
126	1	0	-11.062617	-1.193311	1.762158
127	1	0	-8.812959	-1.453142	2.230412
128	1	0	-8.819562	1.415716	-2.231347
129	1	0	-6.234193	-1.173286	1.844866
130	1	0	-6.239495	1.146659	-1.846469
131	1	0	8.548193	16.939903	-1.327569
132	1	0	7.431476	19.045823	-0.651016
133	1	0	5.932691	19.087564	1.339661
134	1	0	5.580247	16.999273	2.653306
135	1	0	6.722251	14.902430	1.992764
136	1	0	10.332420	15.928655	1.106431
137	1	0	12.710677	16.019210	0.416894
138	1	0	13.491561	14.729008	-1.567156
139	1	0	11.858502	13.361671	-2.861108
140	1	0	9.475232	13.297426	-2.187756
141	1	0	5.800923	14.180697	-0.795159
142	1	0	4.577414	12.050524	-0.782254
143	1	0	8.107868	10.031686	0.625360
144	1	0	9.331109	12.162034	0.612053
145	1	0	-18.953701	-0.674301	1.456285
146	1	0	-20.222322	0.829198	2.962622
147	1	0	-19.503292	3.195571	3.269909
148	1	0	-17.509179	4.041092	2.037129
149	1	0	-16.261134	2.544561	0.507786
150	1	0	-15.188610	0.251137	2.133689
151	1	0	-12.731727	0.279501	2.130210
152	1	0	-12.731258	-0.332783	-2.129780
153	1	0	-15.188234	-0.314324	-2.132382
154	1	0	-18.956474	0.595929	-1.454121
155	1	0	-20.219350	-0.912731	-2.960154
156	1	0	-19.490983	-3.276258	-3.267330
157	1	0	-17.493368	-4.113762	-2.034749
158	1	0	-16.251055	-2.612152	-0.505708
159	1	0	7.239216	-8.589262	0.882648
160	1	0	3.866067	-10.556357	-0.746022

161	1	0	2.296393	-8.868344	-0.927174
162	1	0	6.552999	-6.386664	1.036994
163	1	0	1.373235	-6.418996	-0.745067
164	1	0	4.887129	-4.370555	0.823395
165	1	0	10.400917	-15.884557	-1.102834
166	1	0	12.778278	-15.965726	-0.409027
167	1	0	13.550171	-14.673743	1.577383
168	1	0	11.909086	-13.314142	2.869334
169	1	0	9.526807	-13.259298	2.191664
170	1	0	8.615797	-16.904527	1.328175
171	1	0	7.509084	-19.014748	0.648499
172	1	0	6.014791	-19.061863	-1.345424
173	1	0	5.656724	-16.974577	-2.659135
174	1	0	6.788801	-14.873337	-1.995414
175	1	0	9.383163	-12.122498	-0.607420
176	1	0	8.151110	-9.997290	-0.621645
177	1	0	4.626321	-12.031804	0.777578
178	1	0	5.858596	-14.156865	0.791282

Method: DFT B3LYP 6-31G*
Key word: opt freq
E_{total} (RB3LYP)= -4148.205875 Hartree

5. Cyclic voltammetry studies

Electrochemical studies were performed in an acetonitrile solution containing tetrabutylammonium hexafluorophosphate ($^{n}Bu_4NPF_6$) under an argon atmosphere. Ag/AgCl electrode, glassy carbon electrodes, and platinum electrodes were used as reference electrodes, working electrodes, and counter electrodes, respectively. In total, 5 μl of a 0.8 mg/ml dichloromethane solution of co-oligomer **6** (or **8**) was pipetted and dropped onto a glass-carbon electrode which was allowed to evaporate under the pressure of a washing flask at room temperature (the circular opening of the electrode had a diameter of 4 mm).

References

1. M. Fujinaga, T. Murafuji, K. Kurotobi, Y. Sugihara, Polyborylation of azulenes. *Tetrahedron* **2009**, *Volume 65*, 7115–7121.
2. T.Tsuchiya, T. Hamano, M. Inoue, T. Nakamura, A. Wakamiya, Y. Mazaki. Intense absorption of azulene realized by molecular orbital inversion. *Chem. Commun.* **2023**, *Volume 59*, 10604–10607.
